# Relationship between protein intake and grip strength in qualitative and quantitative aspects among the elderly in Korea: results from the Korea National Health and Nutrition Examination Survey

**DOI:** 10.1186/s12877-023-04016-8

**Published:** 2023-05-26

**Authors:** Mi‑Hyun Kim, Mi‑Kyeong Choi, Yun‑Jung Bae

**Affiliations:** 1 Department of Food and Nutrition, Kongju National University, 32439 Yesan, Republic of Korea; 2 Major in Food and Nutrition, Korea National University of Transportation, 27909 Jeungpyeong, Republic of Korea

**Keywords:** Grip strength, Sarcopenia, Elderly, Protein intake, Legumes

## Abstract

**Background:**

This study investigated the association between quantitative and qualitative protein intake and grip strength (GS) in the South Korean population to explore nutritional management for the prevention of sarcopenia.

**Methods:**

This cross-sectional study was based on data from a nationally representative sample of the South Korean elderly population, consisting of 1,531 men and 1,983 women aged 65 years and older who participated in the Korean National Health and Nutrition Examination Survey from 2016 to 2019. Low GS was defined as GS < 28 kg in men and GS < 18 kg in women. Protein intake was assessed using 1-day 24-h recall, and we analyzed absolute protein intake, protein intake by food source, and protein intake compared to dietary reference intake with per body weight or absolute daily recommended value.

**Results:**

The total and animal protein intake and protein intake from legumes, fish and shellfish were significantly lower in women with a low GS than in those with a normal GS. After adjusting for confounding factors, women who consumed more protein than the estimated average requirement (EAR, 40 g/day for women) were 0.528 times less likely to have low GS than women consuming less protein than the EAR (95% CI: 0.373–0.749), and consuming any amount of protein from legumes were 0.656 times less likely (95% CI: 0.500–0.860) to have low GS than women who did not consume any amount of legume protein.

**Conclusions:**

This study provides epidemiological evidence that adequate protein intake above EAR and protein intake from legumes should be guided for preventing low GS, especially in elderly women.

## Background

Grip strength (GS) is a convenient tool for assessing sarcopenia, a representative aging-related disease characterized by progressive and generalized loss of skeletal muscle mass and strength [1]. Nutritional status is a major determinant of GS in relation to age. Many studies have reported a link between GS and intake of specific nutrients, including proteins [2], vitamin D [3], and antioxidants [4]. Considerable evidence has indicated a positive association between protein intake and muscle strength in combination with exercise or alone among the elderly [2, 5–7]. Based on these results, adequate protein intake is generally recommended for the prevention and management of sarcopenia and frailty in the elderly. However, most of the existing studies have been conducted in Western countries.

South Korea is one of the most rapidly aging countries in the world, and age-related declines in physical performance and increases in chronic diseases have become important public health issues. Although the South Korean diet has been westernized, rice-centered, plant-based meals are still mainstream, and more so in the elderly. According to a study evaluating dietary protein intake and its adequacy among the participants in the 2010–2019 Korean National Health and Nutrition Examination Survey (KNHANES), 34.5% of men and 44.7% of women consumed below than the estimated average requirement (EAR) of protein per kilogram body weight (0.73 g/kg/day) in 2019 [8]. This indicates that the risk of insufficient protein intake among the elderly in South Korea is high. Recently, several studies have reported a relationship between protein intake and GS or sarcopenia in South Korea. Tak et al. [9] reported no association between dietary protein and GS after adjusting for covariates in a study of 1,553 South Korean adults aged ≥ 60 years. While, in a cross-sectional study among 3,634 community dwelling Korean elderly aged $\ge$65 years, inadequate protein intake was positively associated with the risk of a low GS in men [10]. In addition, a previous study on the association between osteosarcopenic adiposity (OSA) and protein intake using the KNHANES 2008–2009 data yielded that elderly over 65 years who consumed less than the recommended nutrient intake (RNI) of 0.91 g/kg/day had a 5.82 times higher risk of OSA than individuals consuming protein equal to or greater than the RNI, after adjusting for confounding factors [11]. Accordingly, the Korean Geriatric Society and the Korean Nutrition Society proposed a protein intake of 1.2 g/kg/day in 2018, which is 31.4% higher than the current recommended level [12].

In addition to the total daily intake of protein, the food source it comes from and its amino acid composition can have an effect on aging-related loss of muscle strength. Sahni et al. [13] reported that total protein and animal protein intake were positively correlated with muscle mass in a cross-sectional study of US Americans. Lord et al. [14] also reported significant correlations between muscle mass and animal protein intake in healthy, normal-weight, sedentary women aged 57–75 years in Canada. In contrast, in a follow-up study of a Chinese population by Chan et al. [15], plant protein intake was significantly associated with a decrease in skeletal muscle mass.

As these existing studies conducted in different countries did not yield consistent results, and the race and dietary patterns were different, a study on the relationship between protein intake and muscle strength in South Korea is needed. This would help derive guidelines for the evaluation and monitoring of various aspects of protein intake in old age and provide clear strategies to prevent aging-related decline in body functions to help the elderly live a healthy life. This study therefore aimed to examine the relationship between GS as a diagnostic indicator of sarcopenia and protein intake, both qualitatively and quantitatively, among elderly South Koreans using representative national survey data.

## Methods

### Data source and study population

In this study, we used raw data from the KNHANES 2016–2019, which provides a representative sample of the South Korean population selected according to a stratified, clustered probability design that has been described in a previous study [16]. The KNHANES is conducted with the approval of the Korea Disease Control and Prevention Agency Institutional Review Board (2018-01-03-P-A and 2018-01-03-C-A), and all participants provide written informed consent before participation. The KNHANES consists of health interviews, health examinations, and nutritional surveys. Of the total of 5,857 participants aged 65 years or older in the KNHANES 2016–2019 data, we excluded participants who had missing data on GS (n = 329); reported implausible energy intakes (< 800 or > 4,000 kcal/day for men, < 500 or > 3,500 kcal/ day for women; n = 173); or had diseases likely to affect GS measurements or protein metabolism that lead to general weakness, including renal failure, cancers, diabetes, and rheumatoid arthritis (n = 1,841). The final study population consisted of 3,514 participants (1,531 men and 1,983 women).

### Grip strength assessment

The GS data were obtained from the KNHANES health examination datasets. GS was assessed using a digital dynamometer (Takei Digital Grip Strength Dynamometer, Model T.K.K.5401, TAKEI, Japan), and participants were asked to perform a GS test alternately with both hands, three times on each side, starting with their dominant hand. In the current analysis, the highest value of measured GS from each participant was used [17], as well as the GS cutoff values recommended by the Asian Working Group for Sarcopenia to determine low GS (< 28 kg for men and < 18 kg for women) [18].

### Dietary assessment

Dietary intake data were extracted from the KNHANES nutrition survey dataset, which was collected using the 24-h recall method for 1 day. To enhance the accuracy of dietary recalls, trained survey staff conducted interviews using two-dimensional food containers and food models, measuring cups and spoons, thickness sticks, 30-cm rulers, and tape measures. In the current analysis, energy intake and percentage of energy intake from macronutrients were examined. Moreover, in order to evaluate protein intake qualitatively and quantitatively, we examined multiple variables related to protein intake. We analyzed protein intake from animal/plant-based sources, protein intake per 1,000 kcal energy intake, protein intake per kg body weight, protein intake from food groups mainly contributing to protein consumption, such as cereals, vegetables, legumes, meats, fish and shellfishes. To compare protein intake with dietary reference intakes (DRI), we analyzed the proportion of participants consuming less protein than the estimated average requirement (EAR, 50 g/day for men, 40 g/day for women), protein intake compared to the RNI (60 g/day for men, 50 g/day for women), and protein intake compared to the protein EAR per kg body weight (0.73 g/kg per day) or protein RNI per kg body weight (0.91 g/kg per day) [19].

### Sociodemographic factors and body mass index

Sociodemographic data were obtained from the KNHANES health interviews. Sociodemographic factors included age, household income, educational level (elementary school graduate or lower, middle school, high school, college or higher), living alone (yes vs. no), smoking status (current vs. non-smoker), alcohol intake (current vs. non-drinker), and physical activity level. For physical activity, we analyzed the proportion of participants who reported engaging in walking exercise (walking for 30 min or more on at least 5 days during the past week) and those who reported engaging in muscle exercise (muscle exercise on 2 or more days during the past week). Body mass index (BMI) data were acquired from the KNHANES health examination datasets, and the degree of obesity was classified according to the Asia-Pacific standard [20].

### Statistical analysis

All statistical analyses were performed using SAS software version 9.4 (SAS Institute, Inc., Cary, NC, USA). Considering the survey design of the KNHANES, clustering variables (PSU), stratifying variables (kstrata), and sampling weights were incorporated into the analyses using the SAS *survey* procedure in this study. According to the cutoff points, the participants were classified into a low grip strength group (low GS) and a normal grip strength group (normal GS). For comparison and analysis, men and women were separately divided into their respective low and normal GS groups. When analyzing sociodemographic factors, nutrient intake, and GS, the mean and standard error were calculated for continuous variables, and frequencies and percentages for categorical variables. Differences between groups were compared using the SAS *surveyreg* and *surveyfreq* procedures. The relationships between qualitative and quantitative protein intake and low GS were analyzed using logistic regression analysis, and the results are expressed as odds ratio (OR) and 95% confidence intervals (CI). As adjustment variables, age, BMI, household income, walking activity, and energy intake were used. In regression analysis, we investigated the association of low GS with total protein intake, quartiles of animal-plant based protein intake and protein intake per kilogram of body weight, protein intake compared to DRI, and protein intake from legumes, meats, fish and shellfishes. In the regression analysis according to protein intake from food groups, cereal and vegetable groups were excluded because there were no participants who did not consume them as Korean diet is traditionally based on grains with vegetable side dishes. The significance level was set at p < 0.05.

## Results

Table 1 shows the participants’ sociodemographic factors. Individuals in the low GS group were older (p < 0.0001 for both men and women), had a lower BMI (p < 0.0001 for men and p = 0.0064 for women), a poorer household income status (p < 0.0001, p = 0.0015), a lower educational level (both p < 0.0001), were living alone (p = 0.0105, p < 0.0001), and engaged less in drinking (p < 0.0001, p = 0.0061), walking (p < 0.0001, p < 0.0001) and muscle exercise activities (p < 0.0001, p = 0.0004).

**Table 1:** Demographic characteristics and their association with low GS among Korean elderly in the KNHANES 2016–2019

	Men (n = 1,531)					Women (n = 1,983)					
	Low GS^1^ (n = 346)		Normal GS^1^ (n = 1,185)		p value	Low GS^1^ (n = 609)		Normal GS^1^ (n = 1,374)		p value	
Age (years)	76.22 ± 0.28		71.63 ± 0.17		< 0.0001	75.63 ± 0.25		71.27 ± 0.15		< 0.0001	
Grip strength (kg)	23.29 ± 0.23		36.20 ± 0.17		< 0.0001	14.62 ± 0.16		22.64 ± 0.10		< 0.0001	
BMI (kg/m^2^)	22.51 ± 0.18		23.89 ± 0.09		< 0.0001	23.78 ± 0.20		24.32 ± 0.10		0.0064	
Distribution											
<23	200(57.85)		435(37.32)		< 0.0001	270(44.92)		481(36.17)		0.0026	
≥23 and < 25	84(25.33)		346(29.53)			126(20.56)		362(26.22)			
≥25	62(16.81)		404(33.15)			213(34.52)		531(37.61)			
Household income											
Quartile 1 (Low)	206(58.54)		385(31.90)		< 0.0001	362(56.46)		633(44.96)		0.0015	
Quartile 2	82(22.84)		393(32.42)			131(23.06)		393(27.55)			
Quartile 3	38(12.15)		224(19.37)			58(11.32)		203(15.43)			
Quartile 4 (High)	17(6.47)		176(16.31)			52(9.16)		141(12.06)			
Education level											
≤ Elementary school	183(55.48)		388(33.17)		< 0.0001	453(80.66)		825(59.72)		< 0.0001	
Middle school	44(16.72)		207(18.26)			45(8.65)		202(15.88)			
High school	50(15.74)		298(26.89)			31(7.10)		197(16.89)			
≥ College	29(12.05)		235(21.68)			16(3.59)		81(7.51)			
Living alone											
Yes	54(14.78)		141(9.65)		0.0105	222(29.40)		366(20.54)		< 0.0001	
No	292(85.22)		1044(90.35)			387(70.60)		1008(79.46)			
Smoking											
Non-smoking	269(80.62)		980(83.00)		0.4071	576(97.71)		1335(97.72)		0.9989	
Current smoking	68(19.38)		196(17.00)			10(2.29)		30(2.28)			
Drinking											
None	185(55.93)		437(35.81)		< 0.0001	496(83.95)		1071(77.54)		0.0061	
Drinking	155(44.07)		739(64.19)			91(16.05)		294(22.46)			
Walking activity											
No	211(67.29)		607(50.97)		< 0.0001	410(76.05)		820(60.50)		< 0.0001	
Yes	94(32.71)		515(49.03)			131(23.95)		484(39.50)			
Muscle exercise activity											
No	266(85.18)		751(66.83)		< 0.0001	524(94.43)		1156(86.68)		0.0004	
Yes	41(14.82)		377(33.67)			21(5.57)		153(13.32)			

Values are expressed as mean ± standard error or frequency (percentage). GS: grip strength, BMI: body mass index

^1^Low GS: Maximum GS of the hands of < 28 kg for men and < 18 kg for women; Normal GS: Maximum GS of the hands of ≥ 28 kg for men and ≥ 18 kg for women

Table 2 presents the respondents’ energy, nutrient, and protein intake. In men, the daily energy intake of the low GS group was 1,846.84 kcal, significantly lower than that of the normal GS group, 1,998.70 kcal (p = 0.0009); in women, there was no significant difference between the low and the normal GS group. In women, the ratios of energy consumed from protein (p = 0.0019), total daily protein intake (p = 0.0024), and animal-based protein intake (p = 0.0027) were significantly lower in the low GS group than in the normal GS group. Protein intake per 1,000 kcal of energy intake was also significantly lower in the low GS group than in the normal GS group among women (p = 0.0010). However, there was no significant difference in protein intake per kilogram of body weight between the groups. In both men and women, the low GS group had a significantly higher proportion of respondents who consumed less protein than the EAR (p < 0.0001), less than the RNI (p < 0.0001), less than 75% of the RNI (p < 0.0001), less than the EAR per kg body weight, 0.73 g/kg (p = 0.0014, p = 0.0003), less than the RNI per kg body weight, 0.91 g/kg (p < 0.0001, p = 0.0012), and less than the protein intake 1.20 g/kg (p = 0.0007, p = 0.0280). Also, in women, the mean protein intake from legumes (p = 0.0008), and fish and shellfish (p = 0.0095) were significantly lower in the low GS group than in the normal GS group. And, in men, the mean protein intake from cereals (p = 0.0466), vegetables (p = 0.0357), and fish and shellfish (p = 0.0371) were significantly lower in the low GS group than in the normal GS group. Meanwhile, in women, protein intake from cereals (p = 0.0092) was significantly higher in the low GS group than in the normal GS group.

**Table 2:** Daily energy and protein intakes according toGSamong Korean elderly in the KNHANES 2016–2019

	Men (n = 1,531)					Women (n = 1,983)			
	Low GS^1^ (n = 346)	Normal GS^1^ (n = 1,185)	p value^2^			Low GS^1^ (n = 609)		Normal GS^1^ (n = 1,374)	p value^2^
Energy (kcal)	1846.84 ± 44.51	1998.70 ± 32.77	0.0009			1475.16 ± 31.76		1513.35 ± 21.4	0.2359
Energy distribution (%)									
Carbohydrate	70.11 ± 0.72	69.27 ± 0.41	0.2724			73.04 ± 0.61		70.49 ± 0.44	< 0.0001
Protein	14.81 ± 0.37	14.65 ± 0.15	0.6814			13.11 ± 0.21		13.80 ± 0.18	0.0019
Fat	15.09 ± 0.50	16.08 ± 0.32	0.0593			13.85 ± 0.47		15.70 ± 0.33	0.0003
Protein intake (g)	65.86 ± 2.10	70.17 ± 1.27	0.0558			47.9 ± 1.23		51.82 ± 0.89	0.0024
Animal protein	28.04 ± 1.66	29.96 ± 0.93	0.2627			17.9 ± 0.94		20.88 ± 0.70	0.0027
Plant protein	37.82 ± 1.21	40.21 ± 0.82	0.0576			30.00 ± 0.73		30.94 ± 0.52	0.2096
Proportion for protein source (%)									
Animal protein	37.22 ± 1.45	38.58 ± 0.82	0.3753			32.8 ± 1.21		36.62 ± 0.85	0.0041
Plant protein	62.78 ± 1.45	61.42 ± 0.82	0.3753			67.2 ± 1.21		63.38 ± 0.85	0.0041
Daily protein intake from food group (g)									
Cereals	19.44 ± 0.68	20.95 ± 0.63	0.0466			17.35 ± 0.52		16.03 ± 0.32	0.0092
Vegetables	5.23 ± 0.28	5.85 ± 0.15	0.0357			4.11 ± 0.17		4.45 ± 0.14	0.0785
Legumes	5.96 ± 0.56	5.49 ± 0.28	0.4270			3.21 ± 0.28		4.35 ± 0.24	0.0008
Meats	13.76 ± 1.39	12.67 ± 0.70	0.4394			7.16 ± 0.76		7.91 ± 0.47	0.3244
Fish and shellfishes	9.22 ± 0.92	11.17 ± 0.58	0.0371			5.86 ± 0.47		7.20 ± 0.45	0.0095
Protein intake density (g/1,000 kcal)	35.20 ± 0.84	34.94 ± 0.34	0.7637			32.51 ± 0.52		34.33 ± 0.44	0.0010
Protein intake per body weight (g/kg)	1.04 ± 0.03	1.08 ± 0.02	0.2195			0.89 ± 0.02		0.93 ± 0.02	0.0845
Protein intake compared to DRI									
< EAR^3^	177(50.81)	362(28.99)	< 0.0001			336(54.16)		498(35.59)	< 0.0001
<RNI^3^	230(67.67)	548(45.38)	< 0.0001			451(72.08)		770(55.34)	< 0.0001
< 75% RNI	149(43.02)	266(20.79)	< 0.0001			302(48.24)		423(29.90)	< 0.0001
75–125% RNI	129(36.94)	509(44.30)				219(38.03)		604(44.97)	
≥ 125% RNI	68(20.04)	410(34.91)				88(13.73)		347(25.13)	
<0.73 g/kg^4^	127(35.97)	330(26.15)	0.0014			303(48.19)		534(38.14)	0.0003
<0.91 g/kg^5^	202(57.84)	346(44.48)	< 0.0001			424(67.69)		808(58.62)	0.0012
< 1.20 g/kg	278(80.23)	838(69.98)	0.0007			525(56.59)		1125(82.04)	0.0280
<0.73 g/kg	127(35.97)	330(26.15)	0.0003			303(48.19)		534(38.14)	0.0006
0.73–0.91 g/kg	75(21.86)	208(18.33)				121(19.50)		274(20.48)	
>0.91 g/kg	144(42.16)	647(55.52)				185(32.31)		566(41.38)	

Values are expressed as mean ± standard error or frequency (percentage). GS: grip strength, DRI: dietary reference intakes, EAR: estimated average requirement, RNI: recommended nutrient intake

^1^Low GS: Maximum GS of the hands of < 28 kg for men and < 18 kg for women; Normal GS: Maximum GS of the hands of ≥ 28 kg for men and ≥ 18 kg for women

^2^P-value after adjusting for age, BMI, household income, and walking activity

^3^Protein EAR and RNI were set as 50 g/day and 60 g/day for the elderly men and 40 g/day and 50 g/day for the elderly women

^4^Protein EAR per body weight

^5^Protein RNI per body weight

Associations between low GS and qualitative and quantitative indicators of protein intake are shown in Table 3 ~ 5. A significant association between protein intake and GS in men was found in the crude model and models 1 and 2, but not in model 3 adjusted for energy intake. Alternatively, in women, in adjusted model 3, participants in the upper quartile of the consumption of total protein intake (Q3 OR = 0.521, 95% CI: 0.340–0.796; Q4 OR = 0.429, 95% CI: 0.251–0.735) presented a lower prevalence of low GS. Also, in adjusted model 3, the animal protein intake range Q4 group of elderly women significantly reduced the risk of low GS compared to those in Q1 group (OR = 0.565, 95% CI: 0.383–0.834). Moreover, elderly women consuming more protein than the EAR (40 g/day) were 0.528 times (95% CI: 0.373–0.749) less likely to have low GS than individuals consuming less protein than the EAR. Similarly, low GS was 0.656 times (95% CI: 0.500–0.860) less likely in women consuming any amount of protein from legumes compared to individuals who did not consume any legume protein.

**Table 3:** Prevalence of low GS and associations of dietary protein according to quartiles of protein intake among Korean elderly in the KNHANES 2016–2019

			Prevalence n (%)^1^	Crude model OR (95% CI)	Adjusted model 1 OR (95% CI) ^2^	Adjusted model 2 OR (95% CI) ^3^	Adjusted model 3 OR (95% CI) ^4^
Men							
Total protein		Q1(≤ 44.6 g/d)	146(36.56)	1	1	1	1
		Q2(> 44.6 and ≤ 59.8 g/d)	83(22.91)	0.516(0.357–0.745)	0.547(0.367–0.815)	0.579(0.377–0.889)	0.663(0.424–1.037)
		Q3(> 59.8 and ≤ 81.7 g/d)	67(15.74)	0.324(0.220–0.477)	0.441(0.289–0.675)	0.543(0.341–0.866)	0.714(0.424–1.202)
		Q4(> 81.7 g/d)	50(12.96)	0.258(0.172–0.389)	0.423(0.267–0.669)	0.559(0.346–0.902)	0.905(0.435–1.885)
		P for trend	< 0.0001	< 0.0001	0.0001	0.0151	0.6361
Animal protein		Q1(≤ 10.3 g/d)	130(33.51)	1	1	1	1
		Q2(> 10.3 and ≤ 21.0 g/d)	98(24.04)	0.628(0.435–0.906)	0.690(0.464–1.025)	0.920(0.601–1.406)	0.993(0.645–1.527)
		Q3(> 21.0 and ≤ 37.7 g/d)	66(17.49)	0.421(0.289–0.612)	0.605(0.397–0.922)	0.743(0.466–1.186)	0.864(0.531–1.404)
		Q4(> 37.7 g/d)	52(13.13)	0.300(0.199–0.452)	0.450(0.287–0.705)	0.627(0.393–0.999)	0.807(0.477–1.364)
		P for trend	< 0.0001	< 0.0001	0.0004	0.0350	0.3624
Plant protein		Q1(≤ 22.3 g/d)	128(31.59)	1	1	1	1
		Q2(> 22.3 and ≤ 35.9 g/d)	86(22.37)	0.624(0.425–0.915)	0.766(0.506–1.159)	0.727(0.472–1.120)	0.851(0.543–1.332)
		Q3(> 35.9 and ≤ 46.1 g/d)	71(18.92)	0.506(0.344–0.744)	0.621(0.407–0.950)	0.632(0.404–0.988)	0.828(0.503–1.362)
		Q4(> 46.1 g/d)	61(15.26)	0.390(0.265–0.575)	0.564(0.363–0.876)	0.624(0.387–1.008)	1.053(0.562–1.974)
		P for trend	< 0.0001	< 0.0001	0.0050	0.0366	0.9092
Protein intake		Q1(≤ 0.69 g/kg/d)	114(28.20)	1	1	1	1
per body weight		Q2(> 0.69 and ≤ 0.95 g/kg/d)	96(24.27)	0.816(0.562–1.184)	0.879(0.589–1.311)	0.949(0.604–1.492)	1.154(0.723–1.843)
		Q3(> 0.95 and ≤ 1.25 g/kg/d)	75(20.09)	0.640(0.442–0.927)	0.681(0.445–1.042)	0.828(0.517–1.324)	1.190(0.687–2.060)
		Q4(> 1.25 g/kg/d)	61(15.57)	0.469(0.313–0.705)	0.554(0.347–0.887)	0.705(0.432–1.152)	1.357(0.670–2.747)
		P for trend	0.0014	< 0.0001	0.0065	0.1485	0.3859
Women							
Total protein		Q1(≤ 32.2 g/d)	221(40.41)	1	1	1	1
		Q2(> 32.2 and ≤ 44.3 g/d)	168(33.78)	0.752(0.552–1.026)	1.003(0.722–1.395)	0.983(0.693–1.394)	0.881(0.603–1.286)
		Q3(> 44.3 and ≤ 59.6 g/d)	123(25.84)	0.514(0.374–0.706)	0.644(0.461–0.901)	0.629(0.448–0.884)	0.521(0.340–0.796)
		Q4(> 59.6 g/d)	97(18.92)	0.344(0.246–0.482)	0.557(0.391–0.793)	0.593(0.409–0.860)	0.429(0.251–0.735)
		P for trend	< 0.0001	< 0.0001	0.0001	0.0007	0.0004
Animal protein		Q1(≤ 6.0 g/d)	220(39.42)	1	1	1	1
		Q2(> 6.0 and ≤ 14.4 g/d)	159(31.37)	0.703(0.518–0.952)	0.892(0.637–1.248)	0.920(0.645–1.310)	0.922(0.646–1.315)
		Q3(> 14.4 and ≤ 25.3 g/d)	131(28.63)	0.616(0.452–0.840)	0.809(0.583–1.121)	0.810(0.580–1.131)	0.817(0.581–1.148)
		Q4(> 25.3 g/d)	99(19.52)	0.373(0.266–0.521)	0.553(0.384–0.796)	0.558(0.383–0.813)	0.565(0.383–0.834)
		P for trend	< 0.0001	< 0.0001	0.0017	0.0019	0.0039
Plant protein		Q1(≤ 21.1 g/d)	195(36.61)	1	1	1	1
		Q2(> 21.1 and ≤ 27.7 g/d)	162(34.13)	0.897(0.655–1.228)	1.045(0.743–1.468)	1.102(0.775–1.566)	1.102(0.759-1.600)
		Q3(> 27.7 and ≤ 36.5 g/d)	127(24.85)	0.573(0.418–0.785)	0.793(0.578–1.088)	0.809(0.578–1.133)	0.809(0.539–1.212)
		Q4(> 36.5 g/d)	125(23.32)	0.527(0.380–0.729)	0.719(0.511–1.012)	0.783(0.547–1.122)	0.783(0.431–1.423)
		P for trend	< 0.0001	< 0.0001	0.0205	0.0690	0.2700
Protein intake		Q1(≤ 0.58 g/kg/d)	205(37.48)	1	1	1	1
per body weight		Q2(> 0.58 and ≤ 0.81 g/kg/d)	156(31.17)	0.755(0.565–1.010)	0.872(0.629–1.210)	0.839(0.589–1.195)	0.831(0.565–1.221)
		Q3(> 0.81 and ≤ 1.07 g/kg/d)	121(24.89)	0.553(0.400-0.763)	0.599(0.422–0.850)	0.579(0.401–0.835)	0.570(0.365–0.890)
		Q4(> 1.07 g/kg/d)	127(25.37)	0.567(0.407–0.789)	0.683(0.476–0.980)	0.730(0.500-1.068)	0.710(0.408–1.235)
		P for trend	0.0005	0.0002	0.0089	0.0373	0.0943

GS: grip strength, OR: odds ratio, CI: confidence interval, BMI: body mass index

^1^p-value for prevalence of low GS were calculated by x^2^-test

^2^P-value after adjusting for age and BMI

^3^P-value after adjusting for age, BMI, household income, and walking activity

^4^P-value after adjusting for age, BMI, household income, walking activity, and energy intake

**Table 4:** Prevalence of low GS and associations of dietary protein according to different recommended protein levels among Korean elderly in the KNHANES 2016–2019

	Prevalence n (%)^1^	Crude model OR (95% CI)	Adjusted model 1 OR (95% CI) ^2^	Adjusted model 2 OR (95% CI) ^3^	Adjusted model 3 OR (95% CI) ^4^
Men					
<EAR^5^	177(33.13)	1	1	1	1
≥EAR	169(16.37)	0.395(0.297–0.526)	0.584(0.428–0.798)	0.703(0.505–0.978)	0.928(0.632–1.362)
P value	< 0.0001	< 0.0001	0.0007	0.0367	0.7012
< 0.73 g/kg^6^	127(27.99)	1	1	1	1
≥ 0.73 g/kg	219(19.68)	0.630(0.474–0.838)	0.677(0.485–0.944)	0.833(0.572–1.213)	1.157(0.754–1.776)
P value	0.0014	0.0016	0.0217	0.3398	0.5042
< 0.91 g/kg^7^	202(26.87)	1	1	1	1
≥ 0.91 g/kg	144(17.67)	0.584(0.445–0.767)	0.645(0.473–0.880)	0.784(0.562–1.094)	1.102(0.714–1.701)
P value	< 0.0001	0.0001	0.0057	0.1514	0.6607
< 1.2 g/kg	278(24.47)	1	1	1	1
≥ 1.2 g/kg	68(15.69)	0.575(0.416–0.794)	0.663(0.459–0.958)	0.802(0.550–1.171)	1.225(0.735–2.041)
P value	0.0007	0.0008	0.0286	0.2535	0.4357
Women					
<EAR^5^	336(39.16)	1	1	1	1
≥EAR	273(23.14)	0.468(0.366–0.597)	0.607(0.469–0.785)	0.605(0.460–0.796)	0.528(0.373–0.749)
P value	< 0.0001	< 0.0001	0.0001	0.0003	0.0004
< 0.73 g/kg^6^	303(34.83)	1	1	1	1
≥ 0.73 g/kg	306(26.16)	0.663(0.528–0.833)	0.750(0.589–0.955)	0.769(0.592–0.997)	0.786(0.569–1.085)
P value	0.0003	0.0004	0.0198	0.0478	0.1428
< 0.91 g/kg^7^	424(32.82)	1	1	1	1
≥ 0.91 g/kg	185(24.82)	0.676(0.532–0.859)	0.726(0.567–0.929)	0.759(0.583–0.988)	0.767(0.544–1.082)
P value	0.0012	0.0014	0.0109	0.0406	0.1301
< 1.2 g/kg	525(30.87)	1	1	1	1
≥ 1.2 g/kg	84(24.00)	0.707(0.518–0.967)	0.892(0.632–1.260)	1.013(0.714–1.437)	1.228(0.819–1.842)
P value	0.0280	0.0298	0.5153	0.9410	0.3199

GS: grip strength, EAR: estimated average requirement, OR: odds ratio, CI: confidence interval, BMI: body mass index

^1^p-value for prevalence of low GS were calculated by x^2^-test

^2^P-value after adjusting for age and BMI

^3^P-value after adjusting for age, BMI, household income, and walking activity

^4^P-value after adjusting for age, BMI, household income, walking activity, and energy intake

^5^Protein EAR and RNI were set as 50 g/day and 60 g/day for the elderly men and 40 g/day and 50 g/day for the elderly women

^6^Protein EAR per body weight

^7^Protein RNI per body weight

**Table 5:** Prevalence of low GS and associations of dietary protein from food group with low grip strength among Korean elderly in the KNHANES 2016–2019

			Prevalence n (%)^1^		Crude model OR (95% CI)	Adjusted model 1 OR (95% CI) ^2^	Adjusted model 2 OR (95% CI) ^3^	Adjusted model 3 OR (95% CI) ^4^
Men								
Legumes		Non-consumer		113(26.60)	1	1	1	1
		Consumer		233(20.52)	0.712(0.545–0.931)	0.723(0.535–0.977)	0.790(0.564–1.107)	0.814(0.583–1.138)
		P value		0.0128	0.0130	0.0345	0.1711	0.2283
Meats		Non-consumer		139(24.32)	1	1	1	1
		Consumer		207(20.72)	0.813(0.616–1.073)	0.926(0.685–1.253)	1.018(0.737–1.407)	1.125(0.803–1.574)
		P value		0.1417	0.1437	0.6192	0.9120	0.4932
Fish and shellfishes		Non-consumer		59(27.04)	1	1	1	1
		Consumer		287(21.33)	0.731(0.509–1.052)	0.772(0.517–1.154)	0.863(0.568–1.312)	0.924(0.604–1.411)
		P value		0.0885	0.0912	0.2069	0.4910	0.7129
Women								
Legumes		Non-consumer		207(36.07)	1	1	1	1
		Consumer		402(27.32)	0.666(0.523–0.848)	0.676(0.520–0.881)	0.648(0.495–0.850)	0.656(0.500–0.860)
		P value		0.0008	0.0010	0.0038	0.0017	0.0023
Meats		Non-consumer		304(32.74)	1	1	1	1
		Consumer		305(27.50)	0.779(0.615–0.988)	0.951(0.734–1.232)	0.911(0.694–1.196)	0.933(0.711–1.224)
		P value		0.0381	0.0395	0.7029	0.5024	0.6168
Fish and shellfishes		Non-consumer		120(37.00)	1	1	1	1
		Consumer		489(28.44)	0.677(0.492–0.930)	0.875(0.630–1.216)	0.943(0.661–1.346)	0.962(0.674–1.375)
		P value		0.0148	0.0161	0.4268	0.7462	0.8334

GS: grip strength, OR: odds ratio, CI: confidence interval, BMI: body mass index

^1^p-value for prevalence of low grip strength were calculated by x^2^-test

^2^P-value after adjusting for age and BMI

^3^P-value after adjusting for age, BMI, household income, and walking activity

^4^P-value after adjusting for age, BMI, household income, walking activity, and energy intake

## Discussion

This population-based cross-sectional study investigated the relationship between protein intake and GS in the elderly South Korean population to explore nutritional management for the prevention of sarcopenia, which becomes more important as the population ages. The current analyses show that elderly women with protein intake higher than the EAR have a significantly lower risk of low GS (OR = 0.528, 95% CI: 0.373–0.749; p = 0.0004) after covariate adjustment, compared to the group with protein intake below the EAR value. In addition, the animal protein intake range Q4 group of elderly women had significantly lower risk of low GS after covariate adjustment, compared to those in Q1 group (OR = 0.565, 95% CI: 0.383–0.834). Elderly women with any amount of protein intake from legumes had also a significantly lower risk of low GS (OR = 0.656, 95% CI: 0.500–0.860; p = 0.0023) after covariate adjustment, compared to the group without protein intake from legumes. These findings thus reveal a significant negative association between protein intake and low GS among elderly women in South Korea.

The Asian Working Group for Sarcopenia (AWGS) proposed a maximum GS of the hands of < 28 kg for men and < 18 kg for women as one of the updated diagnostic criteria for sarcopenia in Asian populations [18]. In our study, KNHANES participants were classified into low and normal GS groups according to this criterion. The percentage of individuals with low GS was 1.4 times as high in women (30.7%) as in men (22.6%). Because muscle strength in one part of the body is closely related to muscle strength in other parts, GS can predict health status related to muscle strength. It has been reported that a decline in muscle strength is an important factor in the deterioration of quality of life because it increases the risk of fragility, fractures, and falls in older individuals [21]. In a prospective population-based cohort study, GS is inversely associated with mortality risk in the elderly, and the association between muscle strength and mortality is stronger in women [22]. Regarding the elderly, a previous study demonstrated that the prevalence of low GS is significantly higher in women than that in men [23], which was similar to this study’s results. Sex difference in the prevalence of low GS is seemingly due to the differences in body composition characteristics and hormone secretion between men and women. Men naturally have higher lean mass than that of women, which may be closely associated with GS. Additionally, it has been shown that women with lower muscle strength than that of men may be more affected by age-related degradation. Age-related decline in muscle strength is thus an important health issue in elderly women, and our finding of a higher number of individuals with low GS in elderly women indicates that the decrease in muscle strength may be especially serious in older women. Since there is a limit to assessing muscle strength using GS only, future research that accurately assesses the decline in muscle strength using various diagnostic indicators in elderly women is necessary to further investigate this issue.

Understanding the determinants of decreased muscle strength is essential for developing novel interventions that effectively maintain muscle health. Factors that influence the decline in muscle strength include exercise and nutrition [24]. Among dietary factors, energy, protein, and antioxidant nutrients have been reported to be related to the loss of muscle strength [2–4, 13]. Dietary protein intake has been the focus of several studies [25, 26], because the amino acids from dietary protein provides are necessary for muscle protein synthesis [27]. In the current study, we evaluated the association of low GS with various criteria of dietary proteins such as quartiles according to intake level, EAR and RNI per day regardless of individual body weight, and EAR and RNI per kg of body weight in Korean elderly aged $\ge$65 years. As a result, protein intake below the daily EAR level (40 g/day for women, 50 g/day for men) was associated with a risk of low GS in elderly women, but not in men. This result is in accordance with earlier reports that higher daily protein intake is positively associated with higher GS in women aged 51 years and older, but not in men of the same age [28]. However, a previous study on Korean elderly showed contradictory results to our current study. Kim et al. [10] reported that the risk of low GS was increased in men whose protein intake was below the EAR level (45 g/day), and that in women, protein intake level according to the EAR was not significantly associated with low GS. The mechanism behind the sex disparity in the association between GS and protein intake, observed in several studies including ours, is unclear but may be explained by sex differences in GS, muscle mass, and hormonal factors [28, 29]. Although there is a small time difference of about 3 years between this study and the study by Kim et al. [10] for the elderly in Korea, the protein intake standards for the elderly in Korea were revised in 2020. The EAR and RNI per body weight were the same at 0.73 g/kg and 0.91 g/kg, respectively, but the daily EAR increased from 45 g/day to 50 g/day due to the change in the standard body weight for the elderly men [19]. There was no change in the daily protein recommended amount for elderly women. Therefore, the results of this study evaluated according to the recently revised protein intake standards and the low GS suggested for Asians are very meaningful in that they review the protein intake standards for the prevention of sarcopenia in the elderly in a timely manner. As can be seen from the analysis of protein intake status (Table 2), the sex disparity in the association between GS and protein intake shown in this study seems to be related to the poor protein intake of Korean elderly women compared to Korean elderly men. In addition, the association between protein intake and GS in men disappeared after adjusting for covariates, including energy intake (Model 3), which suggests that lower energy intake may be associated with a lower GS. In contrast, lower protein intake in women was associated with lower GS regardless of lower energy intake. Energy intake, but not higher muscle strength, was significantly associated with higher muscle mass in a previous study on the elderly [30], after adjusting for sex. Therefore, it is difficult to derive a consistent conclusion on the effect of sex on the relationship between energy intake and GS, and future studies investigating the factors that affect this relationship are required.

Houston et al. [6] recommended that healthy elderly people should consume 1-1.2 g per kg body weight of protein daily to promote muscle protein synthesis and maintain or increase muscle mass. The EAR and RNI of protein for Koreans are based on 0.73 g/kg and 0.91 g/kg of body weight, respectively. By applying the standard body weight to this formula, the EAR and RNI were set as 50 g/day and 60 g/day for the male elderly and 40 g/day and 50 g/day for the female elderly, respectively [19]. In the current study, the status of protein intake was evaluated with the daily EAR and RNI values to which the standard body weight was applied, and the EAR and RNI values considering the body weight of each participant. The findings that protein intake below the daily EAR was associated with a risk of low GS in elderly women, which suggests that protein intake needs to be higher than the EAR to prevent the deterioration of GS, especially in elderly women. In particular, it suggests that meeting the protein EAR per day rather than meeting the protein EAR per body weight is important for the elderly female to prevent low GS. This may mean that the intake of more than a certain amount of protein should be emphasized in order to prevent muscle strength loss even in the case of elderly women who weigh less. In addition, this result can suggest the validity of using the current daily EAR value to guide for the prevention and management of sarcopenia in the Korean elderly women. On the other hand, there was no association between the EAR intake level and low GS in the male elderly in this study, unlike in the female elderly.

The source and amino acid composition of dietary protein can have an effect on aging-related loss of muscle strength. In general, animal protein, which contains sufficient essential amino acids, has been shown to have a better preventive effect on the decline of muscle strength than plant protein. However, as there are also studies [13] showing different results, the relationship between muscle strength loss and protein intake may differ depending on specific food sources and subpopulations. In our analyses on the associations between protein from different food sources, such as meat, fish, and legumes, and GS, only protein intake from legumes showed a significant negative association with the risk of low GS, and only in elderly women. These results can be explained by the role of legumes in the Korean diet characterized by plant foods, where legumes represent a major source of plant protein, their intake is high, and sources of intake, such as beans, tofu, and fermented soybean products, vary. Legume proteins are reported to be of excellent quality because they contain as many essential amino acids as animal proteins [31]. In addition, legumes contain many isoflavones similar to estrogen and female sex hormones, and they are therefore recommended for skeletal muscle health in postmenopausal women [32]. A longitudinal study should be conducted to determine whether soy protein intake has a preventive effect on decreases in GS or muscle strength in elderly women.

Our study has some limitations that one should consider when generalizing its results. This study has a cross-sectional design and used national survey data characteristic of the South Korean population; these limits us in drawing conclusions and applying our findings to other populations. Also, cross-sectional nature of this study does not allow us to determine casual relationships between various risk factors and low GS. The method of measuring muscle strength loss used in the KNHANES has never been standardized, and previous studies have applied it differently. There is, however, a limit to the conclusions one can draw about muscle strength on the basis of GS measurements, and future studies applying a sensitivity evaluation of GS that more clearly reflects muscle strength are therefore needed. The major strengths of this study include the use of a relatively large nationally representative sample and detailed quantitative and qualitative data on protein intake, which allowed us to draw very practical conclusions that can help prevent the decline in muscle strength in elderly people.

## Conclusions

In conclusion, presently, protein intake above the EAR significantly lowered the risk of low GS in elderly women, and protein intake from legumes and animal source was negatively associated with the risk of low GS. These results may serve as a foundation for planning and implementing nutritional intervention programs for the prevention and management of sarcopenia in elderly women with low protein intake. Additional follow-up studies on the various dietary factors that can improve GS are needed in the future.

## Data Availability

Data used in this study is publicly available for research purposes in a de‑ identified fashion at the official website of KNHANES: https://knhanes.kdca.go.kr/knhanes/eng/index.do.

## Abbreviations

GS: grip strength
KNHANES: Korea National Health and Nutrition Examination Survey
RNI: recommended nutrient intake
OSA: osteosarcopenic adiposity
EAR: estimated average requirement
BMI: body mass index
OR: odds ratio
CI: confidence interval
AWGS: Asian Working Group for Sarcopenia
